# Integrated Metabolomics and Amplicon Sequencing Reveal Microbial Succession and Metabolite Dynamics During *Sojae Semen Praeparatum* Fermentation

**DOI:** 10.3390/foods15203761

**Published:** 2026-10-21

**Authors:** Kaili Yan, Na Wang, Shuo Fan, Zehua Chang, Zheng Li, Liuhui Zhao, Yixuan Wang, Xinru Song, Yazhu Yang, Shuai Du, Xinfang Xu, Xiangri Li

**Affiliations:** 1 School of Chinese Materia Medica, Beijing University of Chinese Medicine (Liangxiang Campus), Beijing 102488, China; yankaili9608@163.com (K.Y.);; 2 Traditional Chinese Medicine Processing Technology Inheritance Base of National Administration of Traditional Chinese Medicine, Beijing 102488, China; 3 Beijing Key Laboratory for Quality Evaluation of Chinese Materia Medica, Beijing University of Chinese Medicine, Beijing 102488, China

**Keywords:** *Sojae Semen Praeparatum*, fermentation, untargeted metabolomics, microbial succession, 16S rRNA, ITS

## Abstract

*Sojae Semen Praeparatum* (SSP) is a traditional fermented soybean product with both dietary and medicinal uses in China. However, the dynamic metabolic changes and microbial community succession during SSP fermentation remain insufficiently characterized. In this study, untargeted metabolomics based on UHPLC-HRMS (Ultra-high-performance liquid chromatography-high-resolution mass spectrometry) was integrated with 16S rRNA gene and ITS amplicon sequencing to investigate metabolite remodeling, bacterial and fungal community succession, and their potential associations during SSP fermentation. Clear stage-dependent separation of metabolite profiles was observed, and 22,343 metabolic features were detected, of which 4281 were putatively annotated. Differential metabolites were mainly assigned to amino acids, dipeptides, lipids, carbohydrates, and isoflavones. Sucrose and other carbohydrates were progressively consumed during fermentation, while N-acetyl amino acids, short dipeptides, proline betaine and a range of lipid metabolites built up towards the later stages. The microbiota did not drift at random: amplicon sequencing resolved a clear successional sequence in both kingdoms. Bacteria converged on genera within the Enterobacteriaceae—mainly *Enterobacter*, *Kosakonia* and *Citrobacter*—whereas the fungi started out dominated by *Rhizopus* and ended in a co-dominant configuration of *Aspergillus*, *Mucor* and several minor taxa. When the two data sets were linked, the dominant taxa were associated with metabolites assigned to nitrogen, lipid and phenolic pathways. Procrustes analysis suggested a potential association between the fungal community and the metabolome, whereas the bacterial association was marginal; however, the difference in coupling strength between the two was not directly tested. These results provide preliminary evidence and a foundation for quality control and process optimization, but require validation across independent batches.

## 1. Introduction

Fermentation is a well-established and widely used traditional processing technology. It is commonly applied in the production of fermented foods and also represents an important processing method in traditional Chinese medicine [1]. In recent years, plant-based fermented foods have received increasing attention because of their potential to improve digestibility, sensory quality, nutritional composition, and functional properties [2,3]. Among legume-based fermented foods, soybean is one of the most important fermentation substrates because it is rich in proteins, lipids, carbohydrates, isoflavones, and other bioactive constituents [4]. During fermentation, enzymes secreted by microorganisms and microbial metabolic activities are associated with the transformation of soybean components, including protein hydrolysis, carbohydrate utilization, lipid remodeling, and the conversion of isoflavone glycosides into aglycones with higher bioavailability. These processes affect flavor formation, changes in metabolite profiles, and the generation of bioactive metabolites [5,6]. Therefore, elucidating the dynamic metabolic changes during soybean fermentation is of great significance for quality evaluation and functional development of fermented soybean products.

**Figure 1:**
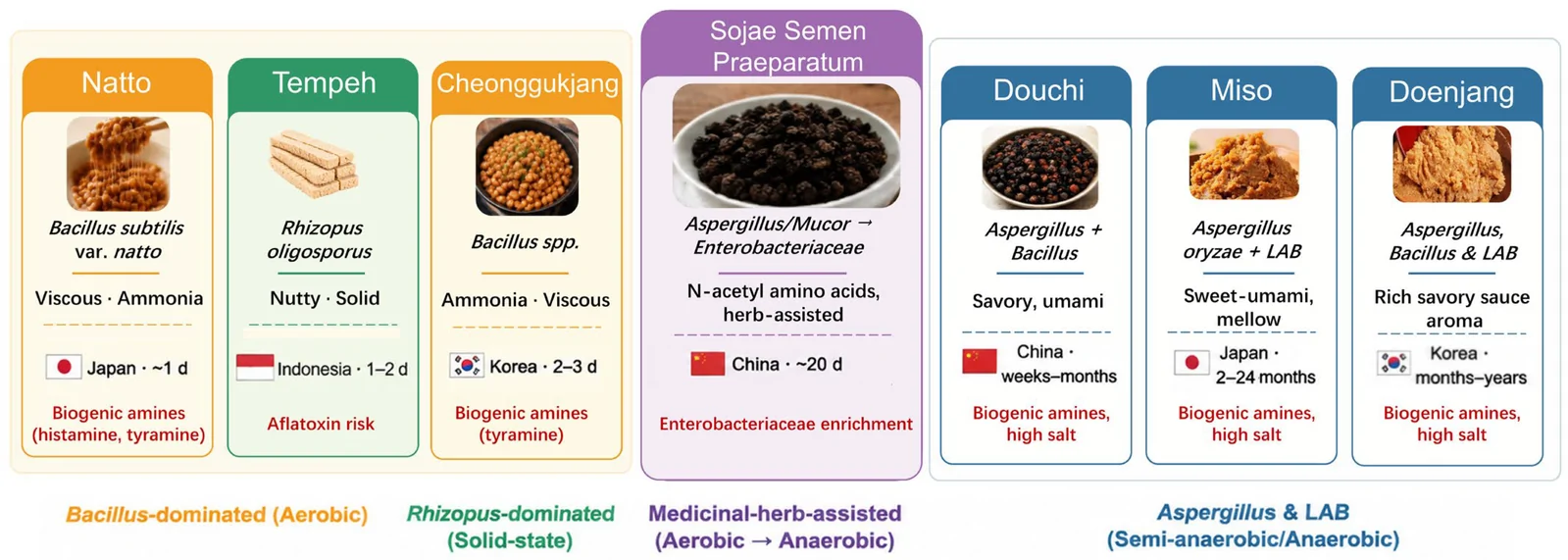
Comparison of representative traditional fermented soybean products.

A variety of representative traditional fermented soybean foods have been developed worldwide, including Japanese natto, Korean cheonggukjang and doenjang, Indonesian tempeh, and various types of Chinese douchi. Although these products are all primarily based on soybean substrates, they differ markedly in raw material composition, use of defined starter cultures, spontaneous fermentation mode, moisture conditions, and processing procedures. Such differences are associated with distinct microbial community structures and divergent transformation pathways for major components [7,8] (Figure 1). In Bacillus-driven systems like natto and cheonggukjang, strong proteolysis is a consistent feature, typically resulting in the accumulation of peptides, free amino acids, and a range of bioactive small molecules [9,10]. Tempeh, in contrast—fermented primarily by filamentous fungi such as *Rhizopus*—is regularly associated with improved protein digestibility, enhanced isoflavone conversion, and noticeable modifications in texture [11,12]. For Chinese traditional douchi and related fermented soybean products, the picture is more complex: these rely on spontaneous, multi-species ecosystems in which both bacteria and fungi contribute jointly to flavour development, metabolite shifts, and final quality differentiation [13,14]. Taken together, these observations underscore that the choice of fermentation mode and the resulting microbial composition are critical determinants of both biochemical transformation trajectories and the ultimate quality attributes of fermented soybean foods. In recent years, the widespread adoption of multi-omics techniques has brought integrated metabolomics and amplicon sequencing to the forefront of fermented food research [15,16], offering a powerful framework for uncovering the mechanisms behind quality formation and for evaluating the functional potential of fermented soybean systems [17].

*Sojae Semen Praeparatum* (SSP) is a unique fermented soybean product in China with both medicinal and dietary uses [18,19]. It is included in the Pharmacopoeia of the People’s Republic of China and has also been consumed as a traditional condiment and functional food in some regions of China. SSP is prepared mainly from black soybean (*Glycine max* (L.) Merr.) through steaming followed by spontaneous fermentation [20,21]. Unlike common fermented soybean foods, the fermentation process of SSP involves herbal decoction-assisted pretreatment and the presence of botanical adjuvants during fermentation [22,23]. Therefore, the metabolic transformation pattern and microbial dynamics of SSP differ from those of conventional fermented soybean products [24].

Although the processing technology and chemical composition of SSP have been reported in several studies [25,26], the systematic coupling relationship between microbial community succession and dynamic metabolite remodeling during fermentation remains poorly understood [27]. In addition, as a medicinal and edible product, microbial safety during fermentation, such as the enrichment of opportunistic pathogens and accumulation of biogenic amines, is directly related to consumer health [28]. However, dynamic safety assessment across the entire fermentation process remains limited [22].

To fill these gaps, untargeted metabolomics (UHPLC-HRMS) (Ultra-high-performance liquid chromatography-high-resolution mass spectrometry) was combined with 16S rRNA and ITS amplicon sequencing in this work, so as to systematically profile changes in metabolites and track the succession of bacterial and fungal communities over the course of SSP fermentation. Correlation analyses were then performed to explore possible links between the dominant microbial taxa and the differentially accumulated metabolites. Specifically, the objectives were to: (1) map the dynamic metabolite landscape across the two fermentation stages (pre-fermentation and post-fermentation) of SSP; (2) characterize the microbial succession pattern observed during herbal decoction-assisted fermentation; and (3) highlight the late-stage enrichment of Enterobacteriaceae as a potential safety concern, thereby offering a new angle for safety evaluation of medicinal and edible fermented products.

## 2. Materials and Methods

### 2.1. Sample Preparation and Collection

#### 2.1.1. Preparation of SSP

SSP was prepared in the laboratory of Beijing University of Chinese Medicine according to the procedure specified under “*Sojae Semen Praeparatum*” in Volume I of the 2025 edition of the Pharmacopoeia of the People’s Republic of China. Black soybean, *Mori Folium*, and *Artemisiae Annuae Herba* were provided by Tongrentang (Beijing, China) and were authenticated as genuine materials by Professor Xiangri Li from Beijing University of Chinese Medicine.

##### Pretreatment of Raw Materials

Surface dust and impurities were removed from black soybeans. *Mori Folium* and *Artemisiae Annuae Herba* were weighed at 100 g each and decocted twice with 10-fold purified water for 1 h each time. The two filtrates were combined to obtain the herbal decoction, and the decocted herbal residues were retained for later use.

##### Mixing and Steaming

A total of 1000 g of soaked and cleaned black soybeans was thoroughly mixed with the herbal decoction of *Mori Folium* and *Artemisiae Annuae Herba*. The mixture was left at room temperature until the decoction had been completely absorbed by the soybeans. The decoction-absorbed soybeans were then transferred to a steamer, steamed, removed, and cooled to room temperature.

##### Pre-Fermentation Stage

The cooled soybeans were placed in sterile fermentation containers, and the surface of the material was completely covered with the decocted residues of *Mori Folium* and *Artemisiae Annuae Herba*. The containers were incubated under constant temperature and humidity conditions at 30 ± 5 °C and 80 ± 5% relative humidity for 5 days until the surface of all soybean grains was uniformly covered with yellow mycelia. After pre-fermentation, the herbal residues were removed, and the materials were rinsed with sterile purified water until no visible yellow mold remained.

##### Post-Fermentation Stage

The rinsed soybean grains were drained and transferred into clean sealed containers. Post-fermentation was conducted under room temperature conditions at 20 ± 5 °C and 70 ± 5% relative humidity for 15 days. Fermentation was terminated when a characteristic aroma was released. The fermented soybean grains were re-steamed for 0.5 h according to the pharmacopoeial requirement and subsequently dried to constant weight to obtain the final SSP product.

#### 2.1.2. Metabolomics Sample Collection

Samples were collected at key fermentation stages: steamed unfermented black soybeans after complete absorption of the herbal decoction, the endpoint of pre-fermentation after 5 days when yellow mycelia had fully developed, and the endpoint of post-fermentation after 15 days of sealed incubation. These samples were designated as YD0, YD1, and YD2, respectively. At each time point, one sample was collected from each of three independent fermentation tanks. All three tanks belonged to the same fermentation batch and were fermented in parallel; therefore, the replicates represent within-batch tank-level replicates (*n* = 3). Samples were immediately frozen and stored at −80 °C until metabolomic analysis.

#### 2.1.3. Microbiome Sample Collection

To capture microbial succession dynamics with higher temporal resolution, samples were collected at eight time points: raw black soybean, fermentation day 0, fermentation day 2, fermentation day 5, after washing on day 5, sealed incubation day 2, sealed incubation day 7, and sealed incubation day 15. These samples were designated as FJ1, FJ2, FJ3, FJ4, FJ5, FJ6, FJ7, and FJ8. At each time point, one sample was collected from each of three independent fermentation tanks, all belonging to the same fermentation batch. Samples were collected into sterile cryotubes, with three within-batch tank-level replicates per group, labeled, and stored at −80 °C for microbial community analysis.

#### 2.1.4. Sample Correspondence

Three representative stages were selected for metabolomics to capture major metabolic transition points, whereas eight time points were used for microbiome analysis to provide finer temporal resolution of microbial community dynamics. For correlation analysis, microbiome samples corresponding to the metabolomic stages were selected according to sampling time. YD0 corresponded to FJ2, both representing steamed black soybeans after complete absorption of the herbal decoction; YD1 corresponded to FJ4, both representing the endpoint of pre-fermentation; and YD2 corresponded to FJ8, both representing the endpoint of post-fermentation (day 20, before final re-steaming and drying). These paired samples were derived from the same fermentation batch and the same sampling time point, and different sample codes were used only for metabolomic and microbiome analyses (Table 1). Owing to the limited number of matched samples, correlation analysis was used only for exploratory hypothesis generation rather than causal inference.

### 2.2. Instruments and Reagents

#### 2.2.1. Instruments

Ultra-high-performance liquid chromatography–high-resolution mass spectrometry (UPLC-HRMS) system: Waters ACQUITY UPLC I-Class liquid chromatography system (Waters, Milford, MA, USA), coupled with a Q Exactive™ HF high-resolution tandem mass spectrometer (Thermo Fisher Scientific, Waltham, MA, USA); electronic analytical balance (Sartorius, Sartorius, Göttingen, Germany, Model BCE124i-ICCN); Nanodrop One nucleic acid analyzer (Thermo Fisher Scientific, Waltham, MA, USA); PCR amplifier (Bio-Rad, Hercules, CA, USA); microcentrifuge (Baygene, Beijing, China, Model BG-Qspin^TM^8482); high-speed refrigerated centrifuge (Eppendorf, Hamburg, Germany, Model 5417R); vortex mixer (Qilinbeier, Nantong, Jiangsu, China); constant-temperature and constant-humidity incubator (Shanghai Yiheng, Shanghai, China, Model BPS-100CA); CNC ultrasonic cleaner (Kunshan Ultrasonic Instrument Co., Ltd., Kunshan, Jiangsu, China, Model KQ-600E).

#### 2.2.2. Reagents and Chemicals

Methanol, acetonitrile, formic acid, and ammonium formate were all of mass spectrometry grade; experimental water was ultrapure water. A microbial genomic DNA extraction kit and agarose were used (Carlsbad, CA, USA).

### 2.3. Non-Targeted Metabolomics Analysis Based on UHPLC-HRMS

#### 2.3.1. Preparation of Test Sample Solutions

Approximately 1 g of each YD0, YD1, and YD2 sample was accurately weighed and placed in a 50 mL conical flask. Then, 25 mL of 75% methanol was added, and ultrasonic extraction was performed for 30 min. A 1 mL aliquot of the supernatant was transferred to a centrifuge tube and centrifuged at 3500 rmp for 5 min. The supernatant was filtered through a 0.22 μm organic-phase membrane, and the filtrate was used as the test solution. Equal volumes of extracts from all samples were pooled to prepare a quality control (QC) sample. One QC sample was injected after every five analytical samples to monitor instrumental stability and chromatographic-mass spectrometric signal drift throughout the analytical run.

#### 2.3.2. Chromatography–Mass Spectrometry Detection Conditions

Metabolite separation was performed on a Waters ACQUITY UPLC I-Class system coupled with a Q Exactive HF high-resolution mass spectrometer. Three within-batch tank-level replicates were analyzed for each group (*n* = 3), and QC samples were inserted throughout the sequence to monitor instrument performance.

##### Chromatographic Conditions

Column: Waters BEH C18 column (1.7 μm, 2.1 mm × 100 mm); mobile phase (positive ion mode): Phase A: 0.1% formic acid in water; Phase B: 0.1% formic acid in methanol; mobile phase (negative ion mode): Phase A: 10 mM ammonium formate in water; Phase B: 10 mM ammonium formate in 95% methanol; gradient elution program: 0–1 min, 2% B; 1–9 min, 2–98% B; 9–12 min, 98% B; 12–12.1 min, 98–2% B; 12.1–15 min, 2% B; flow rate 0.35 mL/min, column temperature 45 °C, injection volume 5 μL.

##### Mass Spectrometry Conditions

An ESI (electrospray ionization) source was used to collect full-scan MS and data-dependent MS/MS acquisition data in positive and negative ion modes, respectively; the scan mass-to-charge ratio range was 70–1050. First-stage mass spectrometry: resolution 70,000, automatic gain control (AGC) 3 × 10^6^, maximum ion injection time 100 ms. The top 3 high-abundance ions were selected based on parent ion response intensity for fragmentation to acquire secondary mass spectra. Secondary resolution 17,500, AGC 1 × 10^5^, maximum injection time 50 ms, and step fragmentation energies (NCE) 20, 40, and 60 eV. Ion source parameters: sheath gas flow rate 40, auxiliary gas flow rate 10; spray voltage: 3.80 kV for positive ions, 3.20 kV for negative ions; ion transfer tube temperature 320 °C, auxiliary gas heater temperature 350 °C.

##### Data Processing and Metabolite Annotation

Chromatographic peak stability and mass spectrometric signal drift were monitored throughout the analytical process. Raw MS data were imported into Compound Discoverer 3.3 (Thermo Fisher Scientific) for peak extraction. The exported results were further imported into metaX for data preprocessing, which included: (1) normalization using Probabilistic Quotient Normalization (PQN) to obtain relative peak areas; (2) batch effect correction using QC-RLSC (quality control-based robust LOESS signal correction); and (3) removal of compounds with a relative standard deviation (RSD, equivalent to the coefficient of variation, CV) > 30% in QC samples to retain only reproducible signals for subsequent analyses.

Metabolite annotation was performed using Compound Discoverer 3.3 in combination with the BGI in-house metabolomics database (BGIHR-PMDB), mzCloud, and ChemSpider. Annotation was based on an accurate precursor mass error (<5 ppm), retention time deviation (<0.2 min), isotope distribution, and MS/MS fragment spectra with a mass error < 10 ppm.

Metabolite identification confidence levels were defined using a customized classification framework from BGI. The identification results were categorized into five levels (Level 1 to Level 5), with confidence decreasing sequentially from Level 1 to Level 5. Level 1: identification against BGI HR-PMDB, where the precursor ion, MS/MS spectrum (i.e., fragment ions), and retention time of the detected result match the reference. Level 2: identification against BGI HR-PMDB or mzCloud, where the precursor ion and MS/MS spectrum match, and the MS/MS spectral matching score is ≥ 60. Level 3: identification against BGI HR-PMDB or mzCloud, where the precursor ion and MS/MS spectrum match, but the MS/MS spectral matching score is < 60. Level 4: identification against any database, based solely on the matching of the precursor ion. Level 5: no identification results after database searching. No authentic reference standards were injected for metabolite confirmation in this study.

A quantitative matrix containing metabolite name, m/z value, retention time, and peak area was exported, preliminarily organized in Excel, and imported into SIMCA 14.1 for multivariate statistical analysis.

##### Multivariate Statistics and Screening of Differentially Accumulated Metabolites

Unsupervised principal component analysis (PCA) was performed to visualize intragroup clustering and intergroup separation among YD0, YD1, and YD2 samples. Because each group contained only three within-batch tank-level replicates and all samples were derived from a single fermentation batch, supervised orthogonal partial least squares discriminant analysis (OPLS-DA) models were considered prone to overfitting, and variable importance in projection (VIP) values were therefore considered less reliable. Accordingly, univariate statistical testing was used as the main approach for differential metabolite screening.

Three pairwise comparisons (YD1 vs. YD0, YD2 vs. YD0, and YD2 vs. YD1) were constructed, and t-tests were performed at the tank level to obtain raw *p* values. Fold changes were calculated and log_2_-transformed, and metabolites with |log_2_FC| ≥ 0.263 and raw *p* < 0.05 were considered differentially abundant. Because the three replicates per group represent within-batch tank-level replication from a single fermentation batch, the reported *p* values describe within-batch (tank-to-tank) variation and should be interpreted as descriptive rather than as formal inference across independent fermentation batches.

For the subsequent analysis of metabolic dynamics during fermentation, thirty representative differential metabolites were selected from this set. The selection was made by ranking |log_2_FC| across all pairwise comparisons and ionization modes, then removing duplicate entries. This strategy was intended to reduce the risk of overfitting that can arise from small-sample models, and it gave more stable and interpretable results.

### 2.4. Amplicon Sequencing Analysis of Bacterial and Fungal Communities

#### 2.4.1. Sample DNA Extraction

Total genomic DNA was extracted using an ALFA Soil DNA Extraction Kit, and DNA purity and concentration were measured using a Nanodrop One (Thermo Fisher Scientific, Waltham, MA, USA).

#### 2.4.2. PCR Amplification and Sequencing

Bacterial and fungal community compositions were analyzed by high-throughput sequencing. The bacterial 16S rRNA gene V3–V4 region was amplified using primers 338F (ACTCCTACGGGAGGCAGCA) and 806R (GGACTACHVGGGTWTCTAAT) (~470 bp). The fungal ITS region was amplified from genomic DNA using barcode-tagged primers ITS1F (CTTGGTCATTTAGAGGAAGTAA) and ITS2 (GCTGCGTTCTTCATCGATGC), targeting the ITS1 region (250–400 bp). PCR was performed using a Premix Taq (TaKaRa) system (2× Premix Taq 25 μL, forward and reverse primers 1 μL each (10 μmol/L), template DNA 50 ng, and ultrapure water to a final volume of 50 μL). The PCR program was as follows: initial denaturation at 94 °C for 5 min; 30 cycles of denaturation at 94 °C for 30 s, annealing at 52 °C for 30 s, and extension at 72 °C for 30 s; followed by final extension at 72 °C for 10 min and storage at 4 °C. PCR products were examined by 1.5% agarose gel electrophoresis, quantified using GeneTools Analysis Software (version 08-3d.3; Synoptics Ltd., Cambridge, UK), and pooled in equal masses. The pooled products were recovered using an E.Z.N.A.^®^ Gel Extraction Kit (Omega Bio-tek, Norcross, GA, USA) and eluted with TE buffer.

The purified products were used to construct sequencing libraries following the standard protocol of the ALFA-SEQ DNA Library Prep Kit (Shenzhen Alpha Biotech Co., Ltd., Shenzhen, China). Library fragment size was evaluated on a Qsep400 system, and concentration was measured using a Qubit 4.0. Libraries (Thermo Fisher Scientific, Waltham, MA, USA) were sequenced on an Illumina PE250 platform, and raw reads were stored in FASTQ format.

#### 2.4.3. Sequencing Data Analysis

No valid fungal ITS amplicons were obtained for the black soybean sample FJ1; therefore, FJ1 was excluded from all fungal community analyses. All conclusions regarding fungi are based on the remaining successfully sequenced samples. Raw paired-end reads were quality-trimmed using fastp (v0.14.1; sliding-window parameters -W4 -M20), and primer sequences were removed using cutadapt (v1.14, -e 0.2) to obtain quality-controlled Clean Reads. The reads were then merged into Raw Tags based on overlap using USEARCH (v10.0.240; -fastq_mergepairs) and filtered again with fastp (v0.14.1) to obtain Clean Tags. After demultiplexing by barcode, the UPARSE algorithm was used to cluster operational taxonomic units (OTUs) at a 97% sequence similarity threshold, and chimeras were removed. Taxonomic annotation was performed using usearch-sintax (v10.0.240), with bacteria compared against the SILVA 16S rRNA database (v138) and fungi compared against the UNITE database (v8.0), using a confidence threshold of 0.8. Sequences with a confidence score below 0.8 were retained as “unclassified.” After annotation, representative sequences originating from chloroplasts and mitochondria (16S amplicons) and sequences that could not be annotated to the kingdom level were removed. Functional annotation was performed using PICRUSt2 (v2.3.0-b) after normalization and comparison against the KEGG database (v94.2). After quality control, 21,560 high-quality sequences were retained per bacterial sample (24 samples in total), and 68,921 high-quality sequences were retained per fungal sample (21 samples in total). To account for differences in sequencing depth, OTU tables were rarefied to an equal number of sequences per sample (21,560 for bacteria and 68,921 for fungi). Alpha diversity indices were calculated based on the OTU table, and community composition was analyzed at the phylum, class, order, family, and genus levels. No negative controls were included in this study; this limitation is acknowledged in Section 4.5.

#### 2.4.4. Data Processing and Statistical Analysis

Alpha and beta diversity analyses were performed using QIIME2 (v2020.11.0). For alpha diversity indices (Observed OTUs, Chao1, and Shannon), the assumption of homogeneity of variance was severely violated (variance ratios exceeded several hundredfold). Therefore, Welch’s ANOVA followed by the Games-Howell post hoc test was used to compare differences among fermentation time points, with *p* < 0.05 considered statistically significant. As a sensitivity analysis, the Kruskal–Wallis test with Dunn’s comparison (Holm adjustment) was also performed to verify the robustness of the results. Other basic data processing was conducted using WPS Office and SPSS 25.0. Correlation analysis between microorganisms and metabolites was performed using the BioinCloud platform (bioincloud.tech). Beta diversity was assessed based on Bray–Curtis distance matrices and visualized using principal coordinate analysis (PCoA). PERMANOVA (adonis2) and homogeneity of multivariate dispersion (betadisper) were performed using the vegan R package (v2.6-4) with 9999 permutations. A significant PERMANOVA result was considered interpretable only when the dispersion test was non-significant (*p* > 0.05).

### 2.5. Correlation Analysis Between Microorganisms and Metabolites

Thirty key differential metabolites, the top 10 bacterial genera, and the top 10 fungal genera by relative abundance were extracted for Spearman correlation analysis. *p* values were adjusted for multiple comparisons using the Benjamini–Hochberg method, applied separately to the bacterial and fungal correlation sets. Correlations were considered significant when |r| > 0.6 and adjusted *q* < 0.05.

To evaluate the overall concordance between microbial communities and metabolite profiles, Procrustes analysis was further performed. Metabolomic data were log_10_-transformed and Z-score standardized before Euclidean distance matrices were calculated. Microbiome data were used to calculate Bray–Curtis distance matrices based on genus-level relative abundance. Principal coordinate analysis (PCoA) was performed separately for the two distance matrices. The resulting PCoA coordinates were then subjected to optimal rotational fitting using the Procrustes analysis module implemented on the Bioincloud platform. The M^2^ statistic was used to quantify the residual sum of squares between the two configurations, with smaller M^2^ values indicating better concordance. Statistical significance was assessed using 9999 permutations [29]. The underlying algorithm was based on the Procrustes and protest functions in the R package vegan v2.6-4.

## 3. Results

### 3.1. Overall Changes in the Metabolite Profile During the Fermentation of SSP

#### 3.1.1. Analysis of Total Ion Chromatograms (TICs) by UHPLC-HRMS

**Figure 2:**
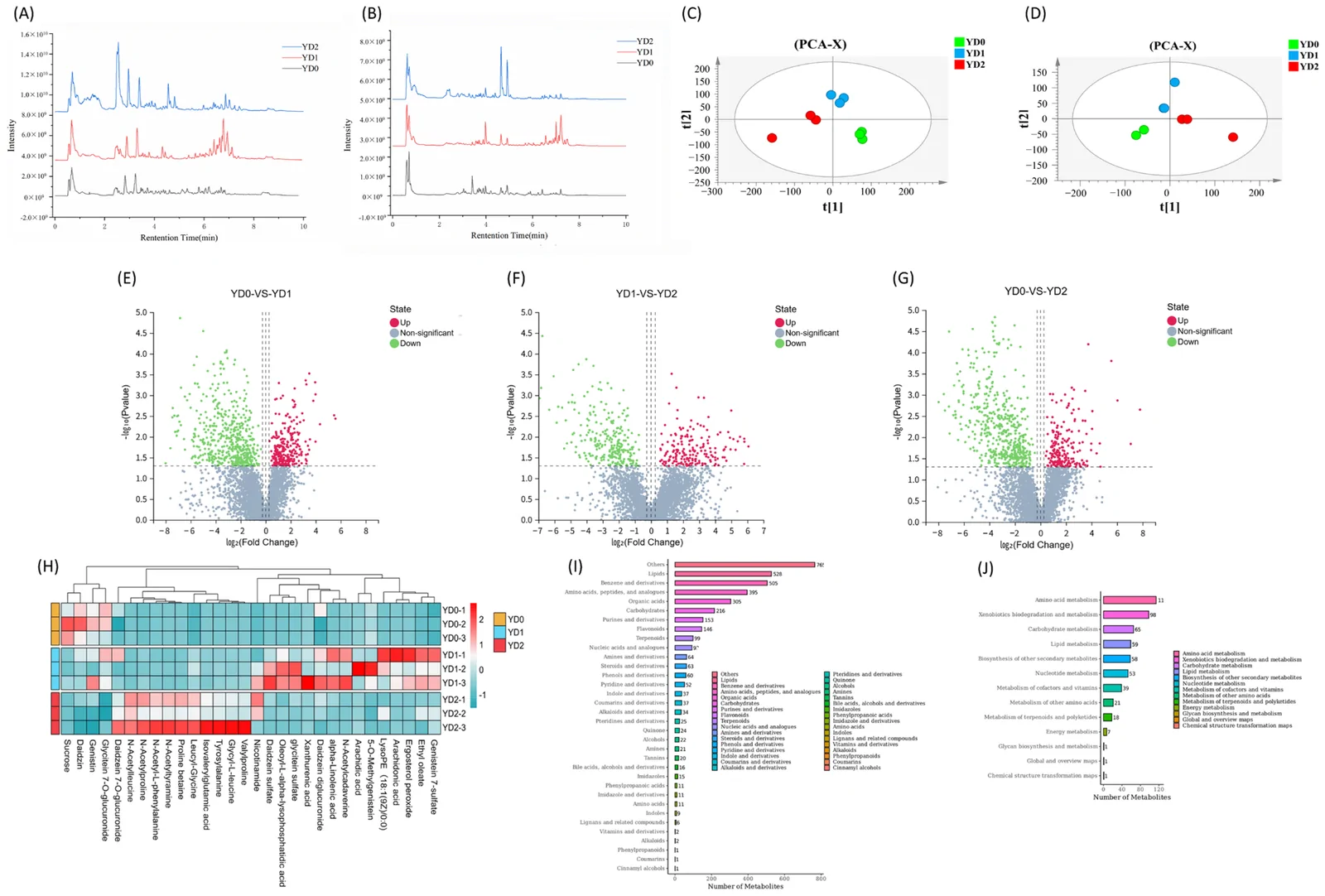
Metabolomic profiles of SSP (*Sojae Semen Praeparatum*) during fermentation. (**A**) Total ion chromatogram (TIC) in positive ion mode; (**B**) TIC in negative ion mode; (**C**) PCA score plot in positive ion mode; (**D**) PCA score plot in negative ion mode; (**E**–**G**) volcano plot for YD1 vs. YD0 (**E**), YD2 vs. YD0 (**F**), YD2 vs. YD1 (**G**); metabolites with |log_2_FC| ≥ 0.263 (equivalent to FC ≥ 1.2 or ≤0.83) and raw *p* < 0.05 are shown in red (up) or green (down). The vertical dashed lines represent |log_2_FC| = 0.263, and the horizontal dashed lines represent *p* = 0.05. (**H**) Heatmap of the top 30 differentially accumulated metabolites; (**I**) chemical class distribution of identified metabolites; (**J**) KEGG pathway classification of the differentially accumulated metabolites (DAMs).

Mass spectrometric data were acquired for all samples in both positive and negative ion modes using UHPLC-HRMS. A total of 22,343 metabolic features were detected, among which 4281 features were putatively annotated. The total ion chromatograms (TICs) acquired in positive and negative ion modes are presented in Figure 2A,B. Good chromatographic peak shapes were observed in all groups. The unfermented raw material group YD0 showed fewer chromatographic peaks and lower signal intensity. After fermentation, the number and intensity of chromatographic peaks increased, and marked changes were observed in retention time distribution and peak patterns. These results indicated that fermentation was accompanied by changes in the overall chemical composition of the samples.

#### 3.1.2. PCA-Based Holistic Metabolomic Pattern Analysis

To get an overall view of the metabolic changes during fermentation, principal component analysis (PCA) was applied to all samples as an unsupervised pattern recognition tool (Figure 2C,D). In the positive-ion mode, PC1 and PC2 captured 27.5% and 21.7% of the total variance, respectively (cumulative 49.2%); in the negative-ion mode, the corresponding values were 28.5% and 20.4% (cumulative 48.9%). The SSP fermentation system is intrinsically complex, containing a large number of compounds such as flavonoids, amino acids and microbial secondary metabolites; as a result, a considerable portion of the metabolic information is spread across the remaining principal components, which explains the relatively modest cumulative contribution of the first two axes. Nonetheless, the PCA score plots from both ionization modes showed a clear separation among the three groups YD0, YD1 and YD2, with samples within each group clustering tightly together, indicating that fermentation was accompanied by stepwise changes in the overall chemical composition. The QC samples also formed a dense cluster, confirming that the instrument performed stably throughout the analysis (Figures S1 and S2).

#### 3.1.3. Statistical Analysis of Differentially Accumulated Metabolites

Metabolites with |log_2_FC| ≥0.263 and *p* < 0.05 were used as the cutoffs to screen for differentially accumulated metabolites, and pairwise comparisons were then performed among the three groups to obtain the counts (Table 2) and to generate volcano plots (Figure 2E–G). For the early fermentation stage (YD1 vs. YD0), a total of 787 differentially accumulated metabolites were identified, of which 510 were upregulated and 277 downregulated. Over the entire fermentation course (YD2 vs. YD0), the total reached 697, with 495 upregulated and 202 downregulated. Even in the mid-to-late stage (YD2 vs. YD1), the comparison still yielded 441 significant metabolites (254 upregulated, 187 downregulated). Across the three comparisons, upregulated metabolites were consistently dominant, ranging from 57.6% to 71.0%. Notably, the highest proportion of upregulated metabolites (71.0%) was observed in the YD2 vs. YD0 comparison, suggesting that a large number of metabolites underwent substantial abundance increases over the entire fermentation process.

#### 3.1.4. Screening of Key Differentially Accumulated Metabolites and Abundance Clustering

To ensure coverage of the major chemical classes, thirty representative differential metabolites were selected by ranking |log_2_FC| values across both positive and negative ion modes, then merging and removing duplicates (Table S1). The abundances of these 30 metabolites were Z-score-standardized and visualized in a hierarchical clustering heatmap (Figure 2H), in which red indicates abundance above the overall mean and blue indicates abundance below the mean, with color intensity reflecting the magnitude of deviation. The heatmap revealed clear differences in metabolic expression patterns among YD0, YD1 and YD2, and within-batch tank-level replicates within each group clustered tightly together, confirming good experimental reproducibility.

The 30 key metabolites were classified into five major categories: flavonoids/isoflavones and derivatives, amino acids/peptides and derivatives, lipids/fatty acids, carbohydrates, and vitamins. In the YD0 raw material samples, sucrose, daidzin, and genistin showed relatively higher abundance, whereas most other metabolites showed lower abundance. In the middle fermentation stage YD1, 5-O-methylgenistein and arachidonic acid were significantly increased. In the final fermentation stage YD2, amino acid derivatives such as N-acetyltyramine and proline betaine were enriched, whereas some metabolites characteristic of the middle stage decreased. These 30 metabolites are referred to as the key differentially accumulated metabolites (DAMs) in the following analyses.

#### 3.1.5. Metabolite Classification and KEGG Pathway Distribution

The chemical classification of all annotated metabolites (Figure 2I) revealed that the largest category was “Others” (761 metabolites), followed by lipids (528) and benzene derivatives (505), with amino acids, peptides, and analogues ranking fourth. The high proportion of the “Others” category reflects the chemical complexity of the SSP fermentation system and suggests that a considerable fraction of the detected metabolites remain unannotated in current databases. This may indicate the presence of fermentation-specific or herbal-derived compounds that warrant further structural identification. The substantial representation of lipids and benzene derivatives is consistent with the rich lipid content of the soybean matrix and the contribution of phenolic compounds from the herbal adjuvants. Meanwhile, the presence of amino acids, peptides, and analogues as a major category aligns with the marked accumulation of protein hydrolysis products observed during fermentation.

KEGG pathway classification (Figure 2J) showed that the three most populated pathways were amino acid metabolism (111 metabolites), xenobiotic biodegradation and metabolism (98), and carbohydrate metabolism (65). The predominance of amino acid metabolism pathways is in line with the sustained increase in free amino acids throughout fermentation, indicating that microbial proteolysis and subsequent amino acid interconversion represent central metabolic activities. The large number of metabolites assigned to xenobiotic biodegradation pathways likely reflects the metabolic transformation of compounds introduced via the herbal decoction, which may provide additional substrates for microbial processing. Carbohydrate metabolism pathways, although ranked third, are consistent with the progressive consumption of sucrose and other sugars, suggesting active glycolysis and related pathways. Importantly, the overall classification and pathway distribution were largely consistent with the category composition of the 30 representative differential metabolites, reinforcing that the major metabolic shifts during SSP fermentation revolve around protein degradation, lipid remodeling, and carbohydrate utilization, alongside the transformation of isoflavonoids and other phenolic compounds.

#### 3.1.6. Dynamics of Free Amino Acids During Fermentation

**Figure 3:**
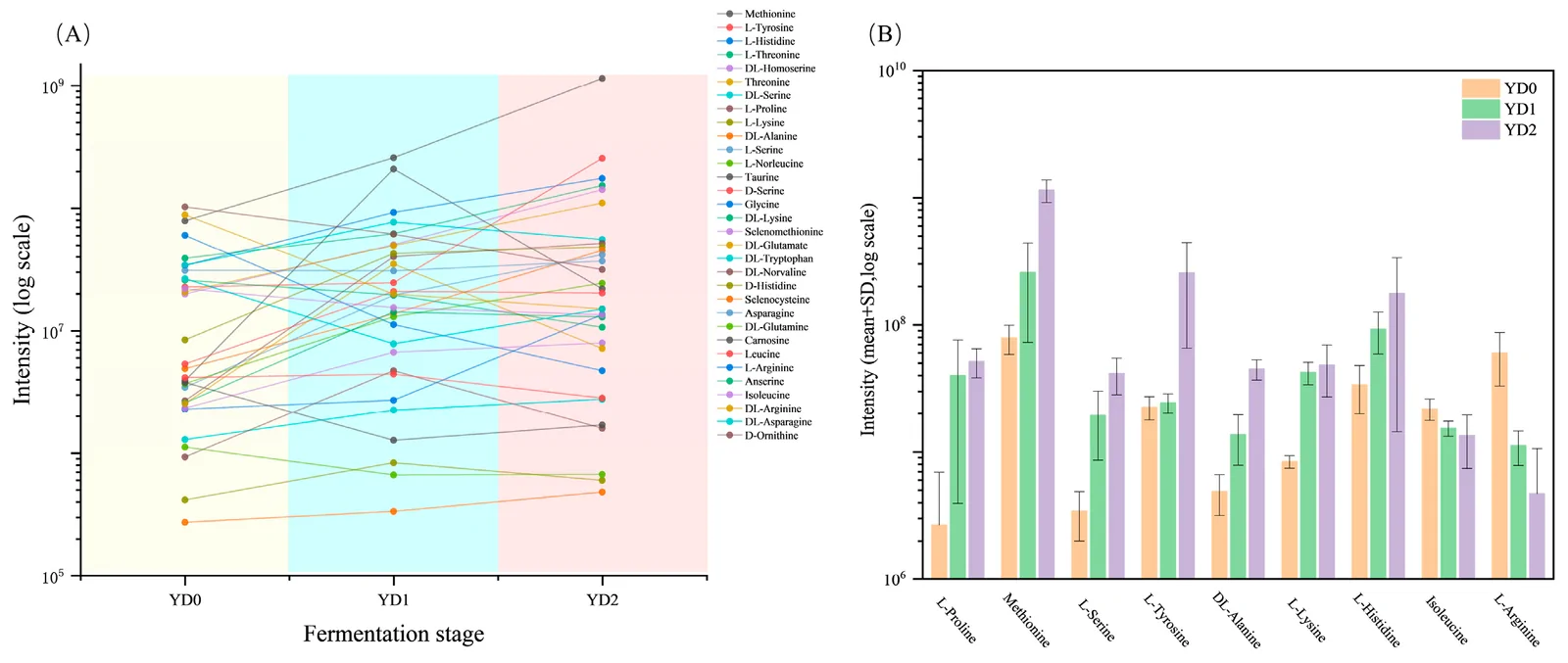
Stage-resolved dynamics of free amino acids during SSP fermentation. (**A**) Line plot showing the abundance changes of all 32 free amino acids across YD0, YD1, and YD2; (**B**) bar charts illustrating the temporal trends of representative free amino acids.

Among the metabolite categories that underwent major remodeling during SSP fermentation, free amino acids—the direct breakdown products of proteins—showed the most consistent and pronounced upward trend. Using the untargeted metabolomics dataset, the stage-specific changes in these amino acids were analyzed across the three fermentation time points (Figure 3). In total, 32 free amino acids were detected, and 22 of them (68.8%) increased in abundance throughout the entire process. This observation points to a progressive, microbe-driven hydrolysis of soybean proteins, which continuously released free amino acids and other nitrogenous compounds.

A closer look at the different fermentation stages revealed a clear division of labor in free amino acid accumulation. The primary stage was dominated by proteolysis; by the end of this phase, L-proline, L-lysine, L-serine, and L-histidine had already built up to substantial levels. The secondary stage then further boosted the levels of methionine, L-tyrosine, L-threonine, glycine, and DL-alanine. Notably, nine amino acids—including L-arginine, DL-arginine, D-ornithine, isoleucine, and leucine—exhibited a net decrease or remained at lower levels over time, most likely because they were consumed as substrates for microbial nitrogen metabolism or converted into peptides and other downstream nitrogen-containing products.

### 3.2. Changes in Microbial Community Composition at Different Stages of SSP FermentationDynamics of Community Composition at the Genus Level

**Figure 4:**
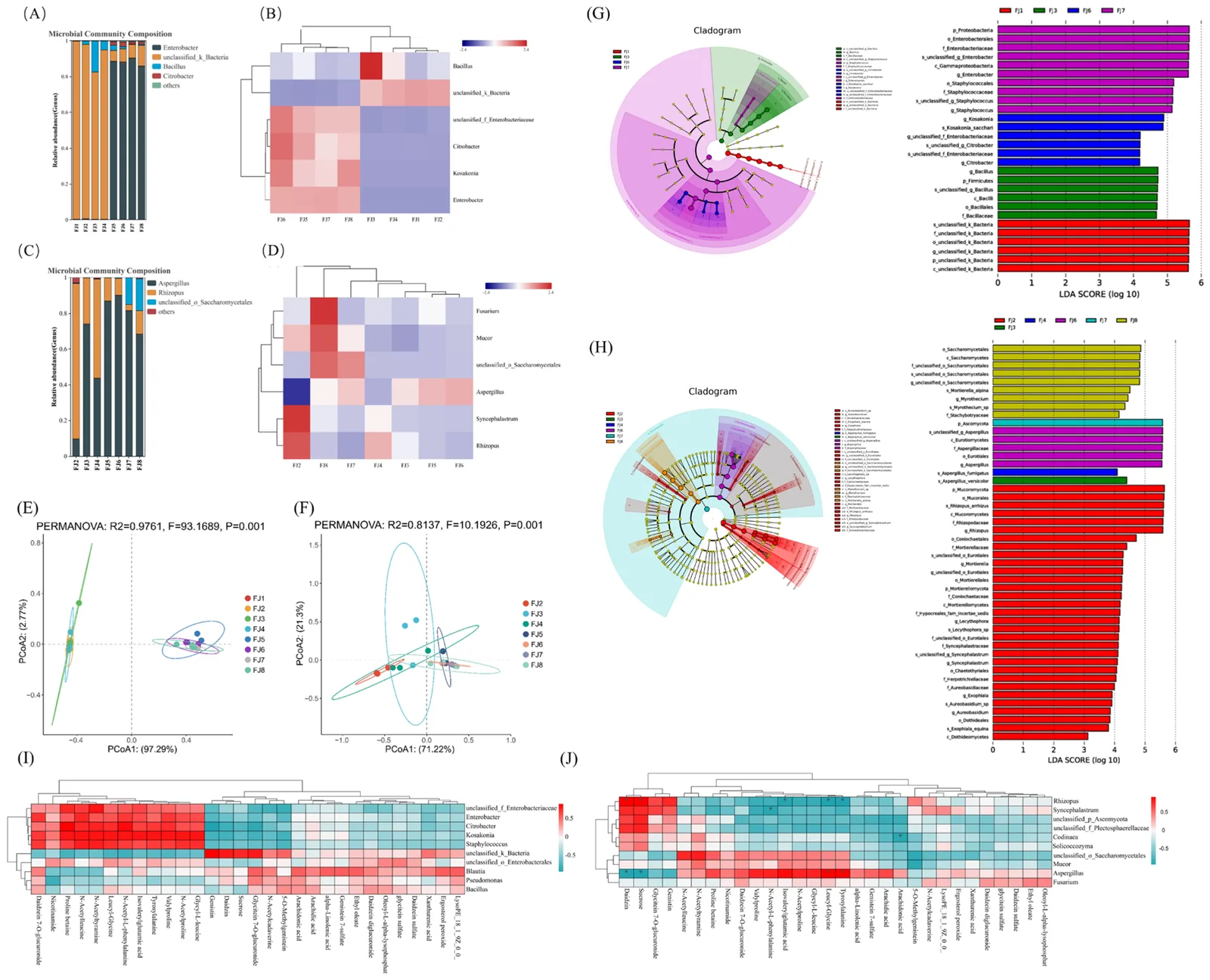
Microbial community succession and its correlation with metabolite dynamics during SSP fermentation. (**A**) Relative abundance of the bacterial community at the genus level (stacked bar chart); (**B**) heatmap of the bacterial community at the genus level; (**C**) relative abundance of the fungal community at the genus level (stacked bar chart); (**D**) heatmap of the fungal community at the genus level. (**E**,**F**) Principal coordinate analysis (PCoA) based on Bray–Curtis distance: (**E**) bacterial community; (**F**) fungal community. (**G**,**H**) LEfSe (linear discriminant analysis effect size) cladograms showing differentially abundant microbial taxa among stages (LDA score > 2.0): (**G**) bacterial species; (**H**) fungal species. (**I**,**J**) Spearman correlation analysis between the top 10 abundant microbial genera and significantly changed metabolites: (**I**) bacteria vs. DAMs; (**J**) fungi vs. DAMs. Notes: Sample FJ1 failed to yield valid fungal sequencing data and was therefore excluded from all fungal community analyses (**C**,**D**,**F**,**H**,**J**). Asterisks indicate Benjamini–Hochberg adjusted q < 0.05. Colors represent Spearman correlation coefficients.

The bacterial community underwent a clear succession over the course of fermentation (Figure 4A,B). In the early stage, unclassified bacteria dominated overwhelmingly. By the middle stage, this group still held a large proportion, but *Bacillus* increased sharply in relative abundance. At the late stage, the community composition changed noticeably: *Enterobacter* became the most abundant genus, and other Enterobacteriaceae-related taxa—such as *Kosakonia* and *Citrobacter*—also rose in parallel.

The fungal community displayed equally pronounced stage-dependent shifts (Figure 4C,D). Early on, *Rhizopus* was the undisputed dominant genus. In the middle stage, *Aspergillus* gained abundance and co-occurred with *Rhizopus*. By the late stage, the fungal assemblage consisted mainly of *Aspergillus*, unclassified Saccharomycetales, and *Rhizopus*.

No valid fungal sequencing data were obtained from sample FJ1; therefore, FJ1 was excluded from all fungal community analyses.

### 3.3. Analysis of Microbial Community Diversity

After quality filtering, removal of chloroplast, mitochondrial, and kingdom-unclassified sequences, and rarefaction to an equal sequencing depth (21,560 sequences per sample for bacteria and 68,921 for fungi), a total of 27 bacterial OTUs and 71 fungal OTUs were retained. The number of OTUs observed per sample ranged from 7 to 17 for bacteria and from 13 to 48 for fungi. Chao1 indices were consistently greater than or equal to the observed OTU numbers in all samples (Table 3). Fungal richness was consistently higher than bacterial richness in all samples with successful fungal amplification, while both communities were dominated by a small number of highly abundant taxa.

#### 3.3.1. α-Diversity Analysis

The observed number of OTUs (richness), the Chao1 index (estimated richness), and the Shannon index (overall diversity) were used to characterize alpha diversity; group-level values (mean ± SD, *n* = 3 tanks) are summarized in Table 3. All indices were calculated from the rarefied OTU tables described in Section 2.4.3.

In all successfully sequenced samples, fungal Chao1 indices were consistently higher than those of bacteria, indicating that the SSP system supported greater fungal species richness. Fungal diversity fluctuated over time. The fungal Chao1 index was highest at FJ2 (45.87 ± 9.07), decreased markedly at FJ3 (28.67 ± 16.15), and then remained relatively stable from FJ4 to FJ8 (31.00–34.70). The Shannon index peaked at FJ3 (0.990 ± 0.123), declined to its minimum at FJ6 (0.386 ± 0.153), and rose again at FJ7 and FJ8 (0.689 ± 0.266 and 0.963 ± 0.412). The decline in Shannon diversity observed at FJ4–FJ6 may be attributable to the washing step after pre-fermentation, which likely removed some surface-attached fungi, followed by community reassembly and recolonization during subsequent fermentation.

For bacteria, the Shannon index rose gradually over the course of fermentation, with only minor dips at FJ4 and FJ7, suggesting that the fermentation environment favored increasing bacterial community diversity. The Chao1 index increased moderately from FJ1 (9.37 ± 2.57) to a peak at FJ6 (17.83 ± 0.83) and then declined slightly, indicating a modest expansion of bacterial taxa in the middle-to-late stage of fermentation. Overall, the bacterial community dynamics were characterized by both taxonomic turnover and a moderate increase in species richness during fermentation.

#### 3.3.2. β-Diversity Analysis

Principal coordinate analysis (PCoA), an ordination technique that projects high-dimensional microbial community data into a lower-dimensional space, was performed to examine β-diversity across different fermentation time points. This approach helps to visualize how samples cluster or disperse, allowing us to infer structural changes in the community under varying fermentation conditions.

The PCoA results based on Bray–Curtis distances are shown in Figure 4, with panel (E) for bacteria and panel (F) for fungi. For the bacterial community, a distinct temporal separation appeared along the PCoA1, which explained 97.29% of the variation: early-stage samples (FJ1–FJ4) clustered on the left side of the plot, while late-stage samples (FJ5–FJ8) grouped on the right. The cluster boundaries were sharp, indicating that fermentation time was the dominant factor driving bacterial community differentiation. PERMANOVA revealed that fermentation stage significantly structured the bacterial communities (R^2^ = 0.9761, F = 93.1689, *p* = 0.001). The dispersion test was non-significant (F = 0.767, *p* = 0.68), confirming that the stage effect was not driven by heterogeneous dispersion.

For the fungal community, PCoA1 and PCoA2 captured 71.22% and 21.3% of the total variation, respectively. Only the FJ2 samples sat apart on the far right; the confidence ellipses for the remaining mid- to late-stage samples (FJ3–FJ8) overlapped extensively, with no clear stage-related separation. PERMANOVA nevertheless revealed a significant effect of fermentation stage on the fungal community (R^2^ = 0.8137, F = 10.1926, *p* = 0.001). The dispersion test was also non-significant (F = 0.77, *p* = 0.64), indicating homogeneity of multivariate dispersion. These results suggest that the fungal community structure changed significantly during fermentation, mainly due to the distinct composition at FJ2, while later stages were more similar to each other.

### 3.4. Linear Discriminant Analysis Effect Size (LEfSe) Differential Species Analysis

To pinpoint microbial taxa that were characteristic of each fermentation stage, LEfSe analysis was performed. This method combines non-parametric tests (Kruskal–Wallis and Wilcoxon rank-sum) with linear discriminant analysis (LDA), and has been widely used to detect taxa showing significant abundance differences among groups [30]. The LDA score threshold was set at 2.0 to define statistically meaningful biomarkers.

For the bacterial community (Figure 4G), marker taxa shifted in a sequential manner over time. Unclassified bacteria were the characteristic taxa at FJ1. At FJ3, *Bacillus*-related taxa became significantly enriched. By the later stages (FJ6 and FJ7), Enterobacteriaceae-associated taxa—particularly *Enterobacter*, *Citrobacter* and *Kosakonia*—emerged as the dominant biomarkers.

For the fungal community (Figure 4H), biomarkers also exhibited stage-dependent succession. *Rhizopus*-related taxa characterized the early stage FJ2. As fermentation advanced, taxa such as *Aspergillus* and unclassified Saccharomycetales were successively enriched from FJ3 through FJ8, and the biomarker composition became more diversified in the late stage. Overall, most fungal biomarkers and their LDA contributions were concentrated in the early and middle stages, whereas their relative abundances dropped markedly in the later phase, suggesting a structural reorganization of the fungal community.

### 3.5. Network Analysis of Correlations Between Microorganisms and Metabolites

Thirty key differential metabolites and the top 10 bacterial and fungal genera by relative abundance were selected for Spearman correlation analysis using the Bioincloud platform. Correlation heatmaps are shown in Figure 4. After Benjamini–Hochberg correction, none of the 300 bacterial correlations remained significant (smallest adjusted *q* = 0.074), whereas 11 of the 300 fungal correlations remained significant (smallest adjusted *q* = 0.024). The strongest fungal correlation was between *Aspergillu* and sucrose (r = −0.93, adjusted *q* = 0.024). Therefore, bacterial associations are presented as exploratory, hypothesis-generating observations, whereas the FDR-significant fungal associations are reported as statistically supported.

#### 3.5.1. Correlation Between Bacteria and Differentially Accumulated Metabolites

At the bacterial level (Figure 4I), Enterobacteriaceae-related genera, including unclassified Enterobacteriaceae such as *Enterobacter*, *Citrobacter*, and *Kosakonia*, were positively correlated with multiple amino acid derivatives, dipeptides, and isoflavone-related metabolites, such as glycyl-L-leucine, valylproline, genistin, and daidzin 7-O-glucuronide. These associations suggested that these bacteria may be involved in the accumulation of protein degradation products and nitrogen metabolism-related metabolites. Such microorganisms may secrete proteases, peptidases, and deaminases to hydrolyze soybean proteins into free amino acids and further transform them into flavor precursor compounds.

*Bacillus*, *Pseudomonas*, and *Blautia* were positively correlated with several lipid- and phenolic-related metabolites, including arachidic acid, alpha-linolenic acid, ethyl oleate, oleoyl-alpha-lysophosphatidic acid, daidzein derivatives, and xanthurenic acid. These results suggested that bacterial community succession may be associated with dynamic changes in nitrogen-containing metabolites, lipids, and phenolic compounds during fermentation.

#### 3.5.2. Correlations Between Fungi and Differentially Accumulated Metabolites

At the fungal level (Figure 4J), *Rhizopus*, *Syncephalastrum*, and several unclassified fungal taxa were positively correlated with early-stage enriched metabolites such as sucrose, daidzin, and genistin, but negatively correlated with most amino acid derivatives and dipeptides. This pattern suggested that these fungal taxa were more closely associated with the substrate metabolic characteristics of the early fermentation stage.

In contrast, *Mucor*, *Aspergillus*, and *Fusarium* were positively correlated with nitrogen-containing metabolites, including N-acetylleucine, N-acetyltyramine, glycyl-L-leucine, valylproline, and tyrosylalanine. These correlations suggested that filamentous fungi may be associated with the accumulation of protein degradation products and amino acid/peptide derivatives.

#### 3.5.3. Comprehensive Analysis

Overall, correlation analysis indicated that SSP fermentation involves both bacteria and fungi. The association patterns between microbial taxa and metabolites differed markedly between these two microbial kingdoms. Enterobacteriaceae-related bacteria were mainly positively correlated with protein degradation-related metabolites, such as amino acids and dipeptides. Filamentous fungi, including *Rhizopus*, *Aspergillus*, and *Mucor*, showed significant associations with soybean glycoside-type isoflavones and lipid metabolites. The bacterial and fungal communities formed a multilayered association network with various metabolites, and temporal microbial succession occurred in parallel with stage-specific changes in metabolite abundance. Together, these processes were associated with the characteristic flavor-related and functional metabolite profiles of SSP.

Because the correlation analysis was based only on matched stage samples and the sample size was limited, the results should be interpreted as hypotheses regarding potential microbe–metabolite associations rather than evidence of causal relationships. These inferred associations require further experimental validation.

#### 3.5.4. Overall Association Between Microbiome and Metabolome

**Figure 5:**
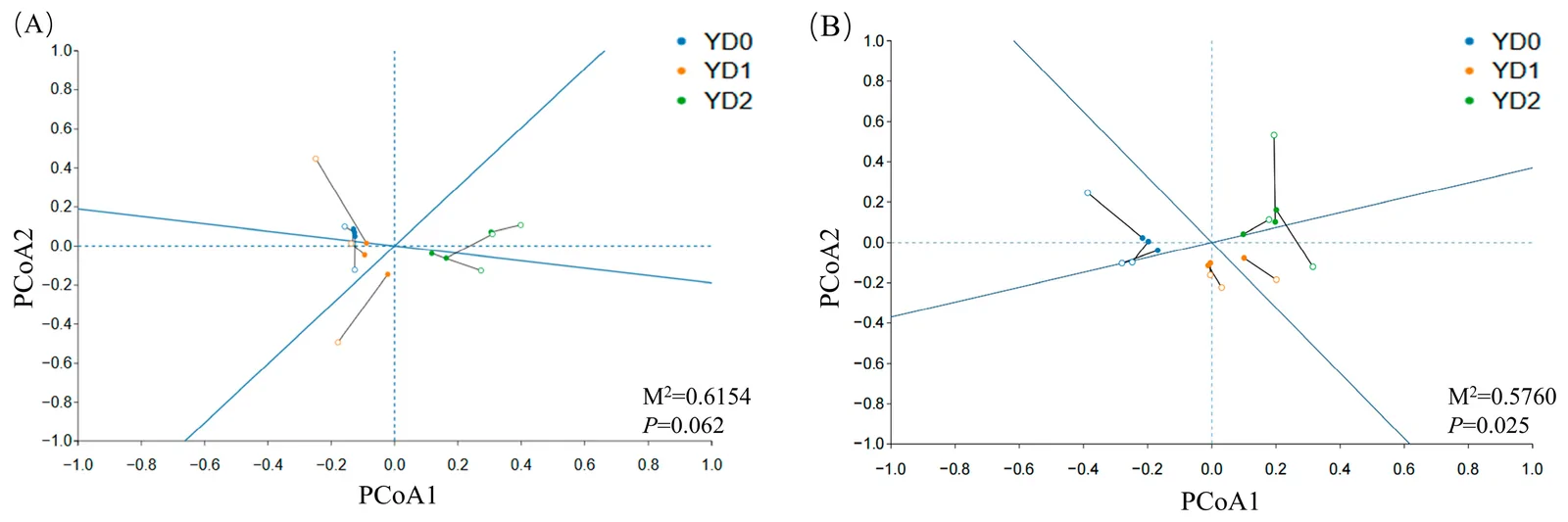
Procrustes analysis between the microbiome and metabolome. (**A**) Bacterial community vs. metabolome; (**B**) fungal community vs. metabolome. Open circles indicate the metabolome PCoA coordinates, solid circles the microbiome PCoA coordinates, solid lines represent the Procrustes rotation axes, and dashed lines link the matched positions of the same fermentation samples. Colors denote fermentation stages: blue for YD0, orange for YD1, and green for YD2. The goodness-of-fit statistics were M^2^ = 0.6154 (*p* = 0.062) for bacteria and M^2^ = 0.5760 (*p* = 0.025) for fungi. All *p* values were derived from 9999 permutations.

To quantify the global concordance between microbial community and metabolomic datasets, Procrustes analysis was performed [31]. The fungal community showed a significant association with the metabolome (M^2^ = 0.5760, *p* = 0.025), whereas the bacterial association was marginal (M^2^ = 0.6154, *p* = 0.062) (Figure 5). It should be noted that this difference in significance does not constitute a formal test of differences in coupling strength between the two kingdoms, and no absolute biomass quantification was performed. Therefore, these results only suggest potential associations and should be interpreted descriptively. This observation is consistent with the Spearman correlation results, in which filamentous fungi—including *Rhizopus*, *Aspergillus*, and *Mucor*—exhibited stronger associations with isoflavone and lipid metabolites.

Notably, the three fermentation stages (YD0, YD1, and YD2) displayed progressive separation in both the microbiome and metabolome PCoA spaces, and the residual connection lines in the Procrustes plots followed consistent directional patterns. This further supports the view that microbial communities and metabolites underwent coordinated succession throughout fermentation.

Taken together, the Spearman correlation and Procrustes analyses captured associations between microbial taxa and metabolite changes during SSP fermentation at both pairwise and global levels. Enterobacteriaceae-related bacteria co-varied mainly with amino acid derivatives and dipeptides, while filamentous fungi such as *Rhizopus*, *Aspergillus*, and *Mucor* showed significant associations with isoflavone and lipid metabolites.

## 4. Discussion

### 4.1. Stage-Specific Metabolite Remodeling and Biological Significance

The metabolomic data showed clear stage-dependent separation among SSP samples during fermentation, confirming that the process substantially reshaped the small-molecule metabolic composition of the soybean matrix. The accumulation of amino acids, dipeptides, and their derivatives may be associated with microbial protease-mediated hydrolysis of soybean proteins [32], while the depletion of sugars such as sucrose probably reflected their consumption as carbon sources by the resident microorganisms [26]. This pattern aligns with the general principle of solid-state fermentation, where fermentable sugars released from plant matrices serve as primary carbon sources driving microbial growth and metabolic activity [33].

A second notable aspect was the accumulation of N-acetyl amino acids and proline betaine during the middle and late fermentation stages. These N-acetylated compounds can be generated by microbial N-acetyltransferases, which transfer acetyl groups from acetyl-CoA to amino acids [34]. Their buildup may reflect microbial nitrogen metabolism or stress-responsive adaptation to changing fermentation conditions, such as shifts in pH, oxygen tension, and nutrient availability. In parallel, the late-stage rise in proline betaine (also known as stachydrine) may be linked to osmotic stress responses, as betaine derivatives are well-recognized osmoprotectants in microbial systems [35].

The isoflavone profile underwent equally pronounced changes. Unfermented black soybeans were dominated by glycosidic forms such as daidzin and genistin. As fermentation advanced, these glycosides were gradually consumed, while free aglycones and secondary modified derivatives—including 5-O-methylgenistein, daidzein sulfate, and daidzein 7-O-glucuronide—appeared or increased in abundance. This pattern suggests that β-glucosidases secreted by the fermentation microbiota may be associated with the deglycosylation of isoflavone glycosides, yielding aglycones with higher bioavailability [36]. Those aglycones may then undergo phase II-like biotransformations such as sulfation and glucuronidation [37]. According to the literature, these fermentation-driven conversions are pharmacologically relevant, since isoflavone aglycones generally exhibit better intestinal absorption and stronger biological activity than their glycosylated precursors [38]. However, the present study did not directly measure bioavailability or intestinal absorption; these nutritional and pharmacological properties are based on the cited literature rather than on direct measurements in this study.

### 4.2. Microbial Community Succession and Its Implications for Fermentation Quality and Safety

Amplicon sequencing revealed marked successional changes in both bacterial and fungal communities during SSP fermentation. In the early stage, *Rhizopus* dominated the fungal community overwhelmingly—a pattern consistent with its well-established role in soybean solid-state fermentation [6]. *Rhizopus* species are known to be well suited to solid substrates, secreting amylases, proteases, and lipases that efficiently break down soybean macromolecules [39]. As fermentation moved forward, taxa such as *Aspergillus* and *Mucor* gradually entered the system, implying that different fungal groups may take on distinct functional roles at successive stages.

The late-stage enrichment of Enterobacteriaceae-related genera—*Enterobacter*, *Kosakonia*, and *Citrobacter* in particular—was probably associated with the environmental shifts that occur when the process switches from aerobic pre-fermentation to sealed post-fermentation. Members of this family are facultative anaerobes and can thrive under low-oxygen conditions. A similar pattern has been observed in Korean *doenjang-meju* fermentation, where anaerobic, facultatively anaerobic, and microaerophilic bacteria were more abundant in inner regions, underscoring oxygen availability as a key determinant of bacterial community structure [40]. The late-stage enrichment of Enterobacteriaceae suggests a potential safety concern, which is discussed in detail in Section 4.5.

Beta diversity analysis further revealed divergent succession patterns between the bacterial and fungal communities. Bacterial communities exhibited a highly ordered temporal progression along the fermentation timeline, whereas fungal communities did not form a clear, stable stage-specific clustering pattern. This divergence implies that the two kingdoms may respond differently to environmental drivers. The orderly replacement of bacterial taxa points to environmental selection as a potentially stronger force in bacterial community assembly. In contrast, the less distinct temporal clustering of fungi suggests that stochastic processes may contribute more substantially to fungal community structure. Similar bacterial-fungal assembly differences have been reported in other solid-state fermentation systems [41]. Quantitative approaches such as null model analysis could help clarify the ecological mechanisms behind these differential responses to the fermentation microenvironment.

### 4.3. Potential Microbe–Metabolite Associations and Mechanistic Inference

Correlation analysis revealed positive associations between Enterobacteriaceae-related genera and a range of amino acids, dipeptides, and N-acetyl amino acids. A plausible explanation is that these facultative anaerobes may utilize small nitrogenous compounds derived from protein hydrolysis as growth substrates—a scenario supported by previous work showing that bacteria in fermented soybean systems can actively participate in amino acid release and conversion via proteases, peptidases, deaminases, and transaminases [42,43].

The correlations involving filamentous fungi—*Rhizopus*, *Aspergillus*, and *Mucor*—were particularly notable for isoflavone, phenolic, and lipid metabolites. These fungi are known to possess strong amylase, protease, lipase, and β-glucosidase activities in fermented soybean matrices [44]. Although their enzymatic repertoires overlap considerably, the correlation data point to a functional division among these genera. Consistent with that, it was found that changes in *Aspergillus* and *Rhizopus* tracked closely with the dynamic profiles of daidzin, genistin, and their derivatives, suggesting a direct role in isoflavone transformation. This is mechanistically plausible, given that *Aspergillus* species are well-documented β-glucosidase producers capable of hydrolyzing isoflavone glycosides to aglycones [45]. Meanwhile, the positive correlation between *Mucor* and nitrogen-containing metabolites points to its possible involvement in protein hydrolysis, which aligns with its known proteolytic activity in sufu fermentation [46]. These distinct association patterns suggest that, although multiple fungal genera may co-participate in substrate degradation, they may differ in the metabolic directions with which they are associated during SSP fermentation.

At the community level, the fungal association was significant while the bacterial association was marginal; however, the difference was not directly tested. Without absolute biomass quantification or a direct test of coupling strength, the relative contributions of fungi and bacteria cannot be compared. The roles of fungi in isoflavone conversion and lipid remodeling therefore remain working hypotheses. The associations reported here describe co-variation rather than causation, and confirming these microbial functions will require integrating metagenomics, metatranscriptomics, and targeted metabolomics in larger cohorts to identify the specific enzymes and pathways involved.

### 4.4. Comparison with Other Fermented Soybean Products

The microbial community structure and metabolic remodeling observed during SSP fermentation showed both shared features and unique characteristics compared with other fermented soybean products. The patterns of protein degradation and carbon source consumption in SSP align well with observations in other fermented soybean products—natto and tempeh, for instance—where similar trends have been reported [47]. However, the underlying microbial drivers and the full spectrum of metabolic outcomes differ markedly. Natto is fermented using a pure culture of *Bacillus subtilis* var. *natto*, and soybean proteins are degraded by microbial proteases during fermentation, resulting in significant increases in all essential amino acids [48]. Although cheonggukjang is a spontaneous fermentation system, *Bacillus* spp. remain the overwhelmingly dominant microorganisms [49]. Metabolomic studies have also confirmed that most amino acids increase during cheonggukjang fermentation. Therefore, *Bacillus* acts as a major functional microorganism throughout these fermentation systems. In SSP, although *Bacillus* was enriched during the middle stage, it did not maintain dominance, and Enterobacteriaceae became the dominant bacterial group in the late stage. This pattern is uncommon in natto or cheonggukjang.

In tempeh fermentation dominated by *Rhizopus*, fungal succession is relatively simple, with *Rhizopus oligosporus* serving as the sole dominant fungus [6]. In contrast, fungal succession in SSP was more complex, shifting from *Rhizopus* dominance to a co-dominant structure involving *Aspergillus*, *Mucor*, and *unclassified Saccharomycetales* [50]. This complexity may reflect the combined effects of spontaneous fermentation, the presence of medicinal plant materials during fermentation, and the two-stage fermentation design.

Compared with long-term anaerobic fermentation systems represented by miso and doenjang, SSP showed a markedly different pattern of metabolic remodeling. Miso and doenjang usually undergo high-salt aging for several months or even years, during which yeasts and halotolerant lactic acid bacteria gradually become dominant functional microorganisms. Their metabolites are mainly sugar alcohols, organic acids, and esters, which contribute to rich fermented aromas [51]. In contrast, the anaerobic post-fermentation stage of SSP does not involve salt addition, and a low-oxygen environment is achieved by sealing the container. The accumulation of amino acid derivatives, such as N-acetyl amino acids, and dipeptides was more prominent in SSP. Therefore, the presence or absence of salt may be a key environmental factor underlying the fundamental differences in bacterial community composition during the post-fermentation stages of SSP and miso/doenjang. In addition, the anaerobic fermentation period of miso and doenjang is much longer than that of SSP, allowing more extensive microbial metabolism and flavor compound accumulation.

The marked accumulation of N-acetyl amino acids and proline betaine in SSP has rarely been reported in other fermented soybean products and may represent characteristic metabolic markers of herbal decoction-associated SSP fermentation [52,53,54]. However, because no decoction-free control was included, the specific contribution of the herbal decoction cannot be isolated in this study.

### 4.5. Implications for Quality Control of SSP Fermentation

The late-stage enrichment of Enterobacteriaceae suggests a potential safety concern that warrants further investigation. However, detection of *Enterobacter* and *Citrobacter* at the genus level does not confirm the presence of pathogenic species; species-level identification and virulence factor analysis would be required to assess actual pathogenicity. In addition, the fungal genera *Aspergillus* and *Fusarium* were present in the later stages of fermentation. Some species within these genera are known to produce mycotoxins, such as aflatoxins and fumonisins, which represent potential safety risks. However, no mycotoxin quantification, viable cell counts, or biogenic amine measurements were performed in this study. Therefore, these observations should be regarded as potential hazards rather than confirmed risks. Future studies should quantify viable Enterobacteriaceae, biogenic amines, and mycotoxins, and perform species-level identification to evaluate the actual safety profile of SSP.

From a quality control perspective, the present findings also offer several actionable points. The endpoints of the two key phases—pre-fermentation and sealed post-fermentation—showed the most pronounced metabolic and microbial shifts, making them logical critical control points for staged monitoring. The progressive depletion of carbohydrate substrates and the gradual build-up of dipeptides and N-acetyl amino acids could serve as chemical indicators of fermentation progress. Meanwhile, the successional dynamics of core fungal genera (*Rhizopus*, *Aspergillus*, and *Mucor*) alongside Enterobacteriaceae-related bacteria might be tracked as microbial markers for real-time process surveillance.

It should be noted, however, that quantitative thresholds for these candidate markers have not yet been defined, and the specific biological roles of many marker taxa in the fermentation system remain incompletely understood. Future work should prioritize targeted quantification of key metabolites, isolation and cultivation of relevant strains, and functional validation experiments, so as to lay the groundwork for a robust quality control framework suitable for industrial-scale SSP production. This study was based on a single fermentation batch. Although three independent containers were used as within-batch replicates, batch-to-batch variation could not be assessed. Therefore, the findings should be considered descriptive and require validation across multiple independent fermentation batches.

## 5. Conclusions

This study integrated untargeted metabolomics and amplicon sequencing to reveal metabolite dynamics and microbial succession during SSP fermentation. Metabolite and microbial profiles shifted progressively in parallel. At the metabolite level, nitrogenous compounds, particularly protein hydrolysis products, accumulated, while carbohydrates were consumed. The microbial community followed an orderly successional trajectory, with Enterobacteriaceae, *Bacillus*, *Rhizopus*, *Aspergillus*, and *Mucor* emerging as principal taxa potentially involved in carbon and nitrogen metabolism. Correlation and Procrustes analyses suggested that the fungal community was more closely associated with the metabolome than the bacterial community. These findings establish a temporal framework linking microbial succession to metabolite turnover, informing the selection of fermentation endpoints and the development of quality control markers for different fermentation stages in SSP production. However, due to the observational design and lack of direct safety or nutritional measurements, these microbial and metabolite associations remain preliminary. Future studies will focus on validation across multiple batches, strain isolation, functional verification, and systematic safety evaluation, aiming to comprehensively assess the quality of SSP and enhance its safety as a health food.

## Figures and Tables

**Table 1:** Sample information and correspondence.

Analysis	Sample Code	Fermentation Stage	Time Point	Description
Metabolomics	YD0	before fermentation	0 d	Unfermented steamed black soybeans
Metabolomics	YD1	end of pre-fermentation	5 d	Yellow mycelium formed
Metabolomics	YD2	end of post-fermentation	20 d	End of post-fermentation (before final re-steaming and drying)
Microbiome	FJ1	raw material	before processing	raw black soybean
Microbiome	FJ2	start of fermentation	0 d	Steamed beans immediately before pre-fermentation
Microbiome	FJ3	pre-fermentation	2 d	Mid-stage of pre-fermentation
Microbiome	FJ4	pre-fermentation	5 d	End of yellow mycelium growth stage
Microbiome	FJ5	after washing	5 d	Washed after pre-fermentation
Microbiome	FJ6	post-fermentation	7 d	Early stage of secondary fermentation
Microbiome	FJ7	post-fermentation	12 d	Mid-stage of secondary fermentation
Microbiome	FJ8	post-fermentation	20 d	End of post-fermentation (before final re-steaming and drying)

**Table 2:** Statistical Table of the Number of Metabolites Showing Differences Across Groups.

Comparison Group	Total	Up	Down
YD1-vs.-YD0	787	510	277
YD2-vs.-YD0	697	495	202
YD2-vs.-YD1	441	254	187

**Table 3:** Alpha Diversity Indices for Bacteria and Fungi Within Sample Groups.

Sample	Bacteria			Fungi		
	Observed OTUs	Chao1 Index	Shannon	Observed OTUs	Chao1 Index	Shannon
FJ1	8.33 ± 2.31 ^a^	9.37 ± 2.57 ^ab^	0.027 ± 0.012 ^b^	—	—	—
FJ2	10.67 ± 1.53 ^a^	12.10 ± 2.65 ^ab^	0.119 ± 0.101 ^ab^	42.00 ± 5.29 ^a^	45.87 ± 9.07 ^a^	0.512 ± 0.184 ^ab^
FJ3	10.33 ± 2.08 ^a^	11.97 ± 1.70 ^ab^	0.332 ± 0.317 ^ab^	21.33 ± 4.51 ^b^	28.67 ± 16.15 ^a^	0.990 ± 0.123 ^a^
FJ4	10.00 ± 2.65 ^a^	11.20 ± 3.20 ^ab^	0.176 ± 0.198 ^ab^	28.00 ± 1.00 ^ab^	32.47 ± 4.39 ^a^	0.829 ± 0.193 ^ab^
FJ5	13.67 ± 0.58 ^a^	13.90 ± 0.72 ^b^	0.480 ± 0.090 ^ab^	17.67 ± 4.04 ^b^	34.70 ± 23.84 ^a^	0.579 ± 0.197 ^ab^
FJ6	15.67 ± 1.53 ^a^	17.83 ± 0.83 ^a^	0.500 ± 0.143 ^ab^	19.00 ± 4.58 ^b^	34.20 ± 6.01 ^a^	0.386 ± 0.153 ^b^
FJ7	12.67 ± 1.53 ^a^	13.50 ± 2.18 ^ab^	0.393 ± 0.027 ^a^	25.00 ± 4.00 ^ab^	31.00 ± 8.35 ^a^	0.689 ± 0.266 ^ab^
FJ8	14.67 ± 1.15 ^a^	14.67 ± 1.15 ^ab^	0.519 ± 0.129 ^b^	33.00 ± 14.73 ^ab^	33.90 ± 13.79 ^a^	0.963 ± 0.412 ^ab^

Note: Values are presented as mean ± SD (*n* = 3 independent fermentation tanks). Different lowercase letters within the same column indicate significant differences among fermentation time points (Welch’s ANOVA followed by the Games-Howell post hoc test, *p* < 0.05). Groups sharing the same letter are not significantly different. Fungal indices for FJ1 are not available (—) because no valid fungal ITS amplicons were obtained. A sensitivity analysis using Kruskal–Wallis with Dunn’s test (Holm adjustment) yielded consistent overall conclusions.

## Data Availability

The raw amplicon sequencing data have been deposited in the NCBI Sequence Read Archive (SRA) under BioProject PRJNA1534174. The untargeted metabolomics raw MS data have been deposited in MetaboLights under accession MTBLS15855. Both datasets are currently private and will be publicly available upon publication.

## Supplementary Materials

The following supporting information can be downloaded at: https://www.mdpi.com/article/10.3390/foods15203761/s1, Figure S1: PCA score plot of the metabolome of SSP samples; the dots represent individual samples, and the QC samples are used to assess the stability of the instrument’s measurements; Figure S2: Analysis of the coefficient of variation (CV) for metabolite quantification based on quality control (QC) samples. The uploaded Excel file contains two sheets: Table S1 lists the 30 key metabolites discussed in the manuscript, and Table S2 provides the complete annotation table for all putatively annotated metabolites (*n* = 4281).

## Abbreviations

The following abbreviations are used in this manuscript:

16S rRNA	16S ribosomal RNA
DAMs	Differentially Accumulated Metabolites
FC	fold change
HRMS	high-resolution mass spectrometry
ITS	internal transcribed spacer
KEGG	Kyoto Encyclopedia of Genes and Genomes
LEfSe	linear discriminant analysis effect size
OTU	operational taxonomic unit
OPLS-DA	orthogonal partial least squares discriminant analysis
PCA	principal component analysis
PCoA	principal coordinate analysis
QC	quality control
SSP	*Sojae Semen Praeparatum*
TIC	total ion chromatogram
UHPLC	ultra-high-performance liquid chromatography
VIP	variable importance in projection
