# Supplementary material for: Integrated Metabolomics and Amplicon Sequencing Reveal Microbial Succession and Metabolite Dynamics During Sojae Semen Praeparatum Fermentation
Source: Foods. 2026 Oct 21;15(20):3761. doi: 10.3390/foods15203761 (PMC13704340; doi:10.3390/foods15203761)
Supplement: Supplementary file 1 [file foods-15-03761-s001.zip › Figure S1.pdf]

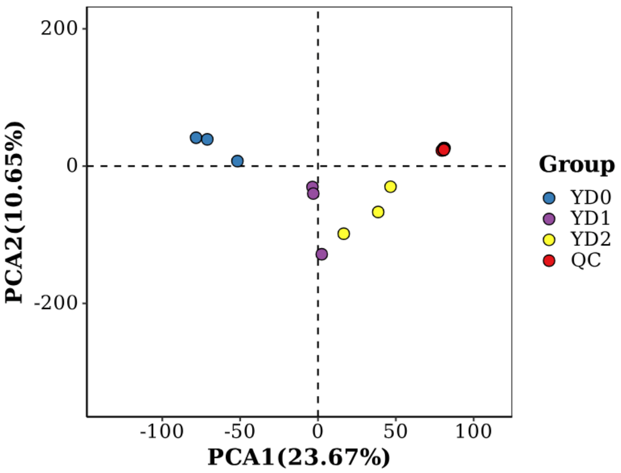

Figure S1. PCA score plot of the metabolome of SSP; the dots represent individual samples, and the QC samples are used to assess the stability of the instrument's measurements.
