# Supplementary material for: Integrated Metabolomics and Amplicon Sequencing Reveal Microbial Succession and Metabolite Dynamics During Sojae Semen Praeparatum Fermentation
Source: Foods. 2026 Oct 21;15(20):3761. doi: 10.3390/foods15203761 (PMC13704340; doi:10.3390/foods15203761)
Supplement: Supplementary file 1 [file foods-15-03761-s001.zip › Figure S2.pdf]

foods

MDPI

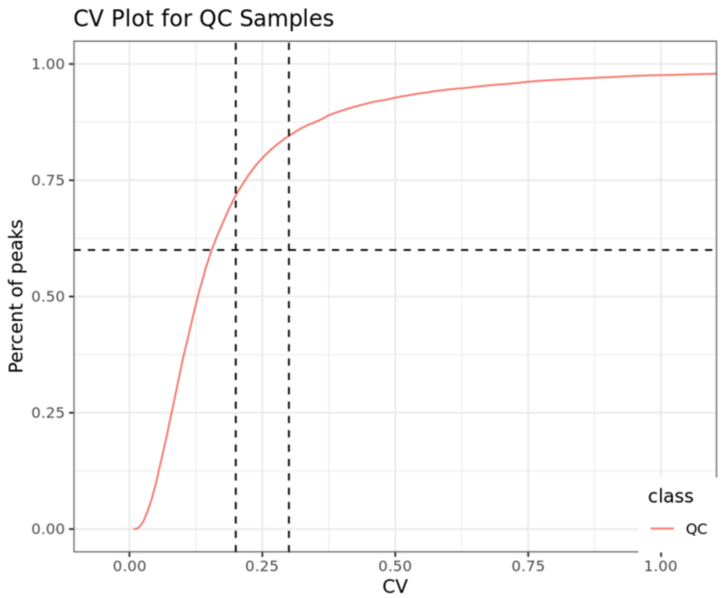

Figure S2. Analysis of the coefficient of variation (CV) for metabolite quantification based on quality control (QC) samples. The coefficient of variation (CV) for each characteristic peak was calculated using the metabolite peak areas from all QC samples; a lower CV value indicates better repeatability in the quantification of that metabolite. In this study, metabolites with a CV ≤ 30% and accounting for ≥60% of the total were considered to meet the overall acceptance criteria for the assay data.
